# Oromaxillofacial Surgery: Both a Treatment and a Possible Cause of Obstructive Sleep Apnea—A Narrative Review

**DOI:** 10.3390/life13010142

**Published:** 2023-01-04

**Authors:** Dinko Martinovic, Daria Tokic, Ema Puizina-Mladinic, Sanja Kadic, Antonella Lesin, Slaven Lupi-Ferandin, Marko Kumric, Josko Bozic

**Affiliations:** 1 Department of Maxillofacial Surgery, University Hospital of Split, 21000 Split, Croatia; 2 Department of Anesthesiology and Intensive Care, University Hospital of Split, 21000 Split, Croatia; 3 Department of Pathophysiology, University of Split School of Medicine, 21000 Split, Croatia

**Keywords:** obstructive sleep apnea, surgical treatment, oromaxillofacial surgery, head and neck surgery

## Abstract

Obstructive sleep apnea (OSA) is a chronic, sleep-related breathing disorder. It is characterized by a nocturnal periodic decrease or complete stop in airflow due to partial or total collapse of the oropharyngeal tract. Surgical treatment of OSA is constantly evolving and improving, especially with the implementation of new technologies, and this is needed because of the very heterogeneous reasons for OSA due to the multiple sites of potential airway obstruction. Moreover, all of these surgical methods have advantages and disadvantages; hence, patients should be approached individually, and surgical therapies should be chosen carefully. Furthermore, while it is well-established that oromaxillofacial surgery (OMFS) provides various surgical modalities for treating OSA both in adults and children, a new aspect is emerging regarding the possibility that some of the surgeries from the OMFS domain are also causing OSA. The latest studies are suggesting that surgical treatment in the head and neck region for causes other than OSA could possibly have a major impact on the emergence of newly developed OSA, and this issue is still very scarcely mentioned in the literature. Both oncology, traumatology, and orthognathic surgeries could be potential risk factors for developing OSA. This is an important subject, and this review will focus on both the possibilities of OMFS treatments for OSA and on the OMFS treatments for other causes that could possibly be triggering OSA.

## 1. Introduction

Obstructive sleep apnea (OSA) is a chronic, sleep-related breathing disorder. It is characterized by a nocturnal periodic decrease or complete stop in airflow due to partial or total collapse of the oropharyngeal tract [1]. These obstructive episodes cause asphyxia, which then stimulates breathing efforts against the collapsed upper airway and subsequent awakening. The etiology of the disorder is still unknown, but it is considered to be multifactorial as a combination of anatomic variations, neuromuscular factors, and genetic predispositions [2]. In adults, OSA is most frequently associated with male gender, obesity, and rising age while in children, it is most commonly associated with enlarged tonsils or adenoids. However, even with today’s technology, many patients with OSA are undiagnosed and untreated, which is a major global healthcare problem since it is well-established that OSA is associated with high cardiovascular morbidity and mortality [3]. Moreover, independently from body weight, patients with OSA more frequently develop metabolic disorders, such as insulin resistance and hyperlipidemia [4].

**Figure 1:**
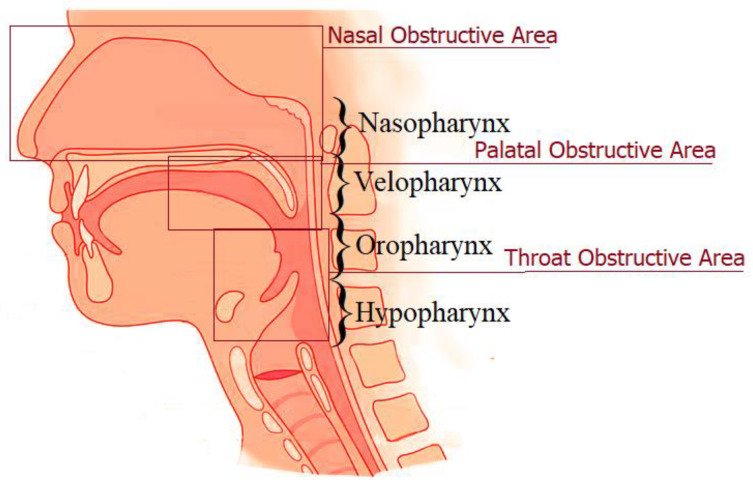
Sagittal view of the upper airway and the places of obstruction in OSA.

There are several treatment modalities for OSA, both conservative and surgical. Continuous positive airway pressure (CPAP) is considered the “gold standard” for the treatment of OSA due to the fact that it can be used for the improvement of sleep-related symptoms and quality of life [5]. On the other hand, since OSA is related to anatomical disturbances of the upper airway, surgical treatment is aimed at reducing the degree of obstruction in the nasal region, nasopharynx, velopharynx, oropharynx, and hypopharynx (Figure 1).

While it is well-established that oromaxillofacial surgery (OMFS) provides various surgical modalities for treating OSA both in adults and children; a new aspect is emerging regarding the possibility that some of the surgeries from the OMFS domain are also causing OSA. The latest studies are suggesting that surgical treatment in the head and neck region for causes other than OSA could possibly have a major impact on the emergence of newly developed OSA [6,7,8,9,10,11]. On one hand, there are oncological surgeries where indications for surgery are of vital importance, but due to tissue loss and reconstruction, they consequently cause anatomical changes and abnormalities. Furthermore, trauma surgery in the OMFS region has its own difficulties, especially regarding maintaining functionality, and in complex fracture cases, it could also be a cause of OSA due to structural alterations. Lastly, there is orthognathic surgery, which represents both a functional and aesthetical component of OMFS with a significant impact on the morphology and anatomy of this specific region. Even though the reasoning behind this possibly higher risk for OSA after OMFS is logical due to anatomical deviations after certain surgical therapies, this subject has become a hot topic only relatively recently. Hence, this review will focus on both the possibilities of maxillofacial surgical treatments for OSA and on the treatments for other causes which could possibly be triggering OSA.

## 2. Oromaxillofacial Surgery as a Cause of Obstructive Sleep Apnea

Recent studies are suggesting that treatment modalities for cancers in the head and neck region, both surgical and conservative, could be a major risk factor for developing OSA [6]. As aforementioned, surgery in this specific region causes structural alterations that could possibly have severe consequences on airflow through the upper airway while it was also shown that radiotherapy causes edema of the soft tissue and consequent change of anatomic relations [6]. Several studies investigated the development of OSA in patients with head and neck cancers [9,12,13,14]. The results of these studies greatly vary due to the number of patients enrolled in the study or to the different treatments for cancer. Nevertheless, all of the studies found that surgically treated patients have a higher prevalence of developing OSA. A study by Loth et al. found that 25.49% of the patients enrolled in their study developed OSA after the treatment of cancer regardless of the type of treatment [12]. Furthermore, Friedman et al. estimated the prevalence of OSA at 72.7% [13]. However, this study had a more heterogeneous population and did not exclude pre-existing risk factors. A study conducted by Qian et al. investigated the prevalence of OSA after cancer therapy, and they found that patients treated with surgery have a higher prevalence of developing severe OSA compared to patients on chemo/radiotherapy. Still, this study has limitations, such as a small sample size, incomplete matching in body mass index, and primary tumor location [14]. Furthermore, Ralli et al. found that the prevalence of OSA in head and neck cancer patients is significantly higher when compared to the general population [6]. However, their study was conducted on all treatment modalities, surgical and nonsurgical. Additionally, even though most of the aforementioned studies focused on surgery as a cause of OSA from the cancer excision point of view, a recent study was conducted on patients who were reconstructed with free flaps in the oral region. They found that even the reconstruction technique could possibly influence the emergence of OSA [9].

It is very important to highlight the fact that not only the surgical treatment of cancer in the OMFS region causes OSA, but the tumor itself has a major impact. When growing to the point that it causes anatomical alterations, it is possible that malignancies could influence the development of OSA. Moreover, tumors are a major limitation of the studies regarding OSA development after OMFS oncological surgeries. This limitation is due to the fact that none of the involved participants had a precancer screening of OSA since most of these patients are first screened after the cancer diagnosis; hence, it is impossible to exclude pre-existing OSA and a tumor is a major bias for interpreting the influence of surgery on OSA. Moreover, head and neck tumors as a cause of OSA are very poorly investigated. Payne et al. found a strong association between OSA and oral cavity cancers before the treatment, but they had a small sample of 17 patients included in the study [15]. Moreover, a case report by Gomez-Merino et al. presented a patient with pharyngeal non-Hodgkin’s lymphoma whose first symptoms were sleep disorders, such as snoring and breathing pauses during sleep [16]. Another interesting case report by Hockstein et al. showed an interesting finding of retropharyngeal lipoma extended from the nasopharynx to the upper mediastinum which also caused symptoms of OSA [17]. Since the tumor was benign and the patient had several chronic diseases that made him high risk for surgery, the only treatment was CPAP. There are several similar cases describing OSA as the primary symptom of cancer [18,19,20]. However, this subject is very scarcely represented in the literature, and future larger prospective studies are needed. Nevertheless, healthcare workers involved in the diagnostics and treatment of OSA should always consider both anamnestic details and potential illness as triggers for newly discovered OSA.

When it comes to OMFS trauma, it is often accompanied by injuries to other parts of the body, and according to some studies, it is involved in up to 25% of polytraumatized patients [21]. The most common cause of these injuries is traffic accidents while other more frequent causes are assaults and falls. Furthermore, injuries in this region can range from simple soft-tissue lacerations to complex bone fractures [22]. Fractures in OMFS have both aesthetic and functional aspects, which makes them even more challenging. As already mentioned, any surgical manipulation in this specific area can lead to both hard and soft tissue impairments and subsequent morphologic alterations, which possibly creates a predisposition for the development of OSA. A recent study conducted by Lupi-Ferandin et al. investigated the prevalence of OSA after surgical treatment of maxillary and zygomatic fractures [11]. They found that 54% of their participants developed OSA in a period of three months after surgery. Furthermore, their study showed that OSA is more common in patients with maxillary fractures (62%) than in patients with zygomatic fractures (38%). Another recent study conducted by El-Anwar et al. also found that OSA is connected with trauma in the OMFS region. Their study investigated mandibular fractures and found that OSA was diagnosed in 35.8% of their patients, all with bilateral fractures [23]. Although both of these studies investigated OSA in OMFS trauma and their results are suggesting a possible association between the development of OSA and OMFS trauma, it must be noted that the data regarding this subject are still too scarce. While it is reasonable to assume that both the bone fractures and consequent surgical therapy could possibly impact OSA, on the other hand, further larger studies are needed to evaluate this issue.

Besides trauma and cancer treatment surgeries, another important segment of OMFS is orthognathic surgery. These OMFS procedures manipulate anatomical relations of the maxilla and mandible in order to achieve certain functional and visual improvements of deformities caused by dentofacial conditions or carried out purely for aesthetic reasons [24]. While these types of surgeries require an advanced level of skills, some effects of these surgeries are expected because of the manipulation of the mandible, anatomical features, and morphology of the upper airway. Several studies found that mandibular setback surgery has an impact on respiratory function, more precisely, a reduction in the retrolingual airway space [25,26]. However, these findings are still controversial. A review by Fernandez-Ferrer et al. analyzed the literature on this topic, and their observations are rather interesting; they found that nasopharyngeal space does not change significantly after maxillary surgery but only after mandibular setback [27]. It was shown that there are significant changes in the volume of space in this area, yet there was no evidence found that confirms the hypothesis that orthognathic surgeries predispose the development of OSA. Another interesting point of view is comparing and analyzing the differences between sexes after orthognathic surgeries. A retrospective study by Dahy et al. found that male patients have a higher risk of developing OSA after mandibular setback surgery while females have a higher risk after bimaxillary surgery [28]. One of the possible explanations for these differences are purely morphological; several studies found that females have smaller pharyngeal airways [29,30,31]. However, considering that this specific area is in a dynamic interaction between muscular activity and anatomic relations, studies found that the female upper airway is more stable [32,33,34].

## 3. Surgical Therapy for Obstructive Sleep Apnea

**Figure 2:**
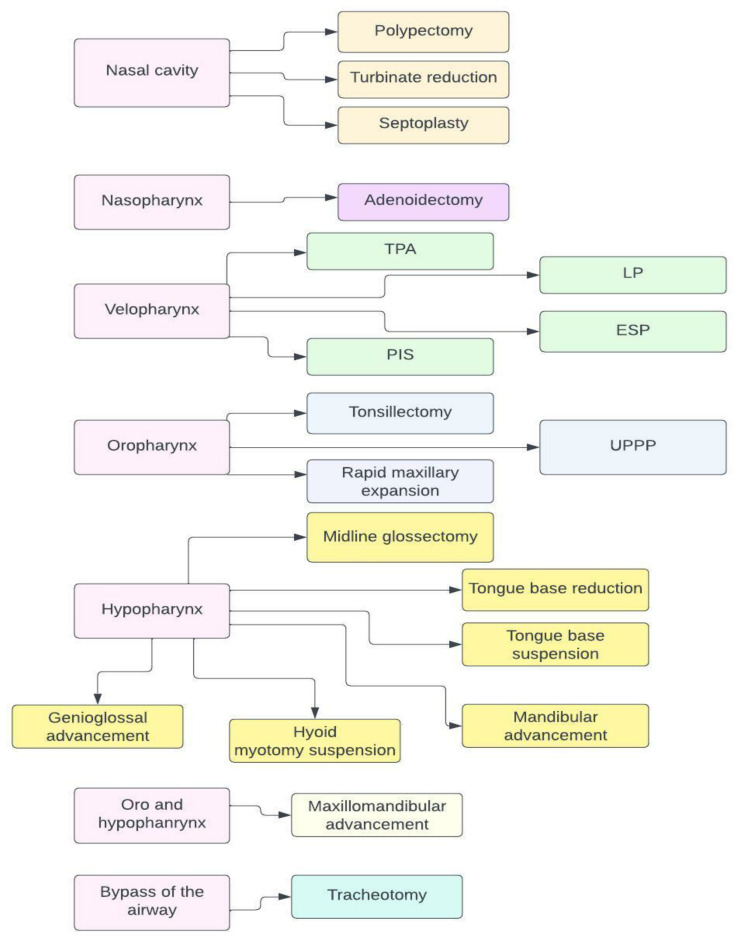
Possible surgical therapies depending on the different level of obstruction. Abbreviations: TPA—trans palatal advancement pharyngoplasty; LP—lateral pharyngoplasty; ESP—expansion sphincter pharyngoplasty; PIS—Pillar implant system; UPPP—Uvulopalatopharyngoplasty.

The anatomical reasons for OSA are very heterogeneous due to the multiple sites of potential airway obstruction. Henceforth, various surgical treatment options have been established, especially aimed at patients who have unsuccessfully undergone positive airway pressure therapy. These surgical treatments aim to alleviate the upper airway obstruction in the nose, nasopharynx, velopharynx, oropharynx, and hypopharynx. Moreover, in the case of a severe OSA, the focus of surgery is on the hypopharyngeal or retrolingual obstruction, which is most commonly associated with an enlarged tongue or maxillar and/or mandibular deficiency (Figure 2). Surgical therapy success is defined as achieving a reduction of the apnea–hypopnea index (AHI) greater than 50% and/or achieving an AHI of less than 20 events per hour [35]. After the procedure, the success rate is based on the polysomnography (PSG) results and the patient-reported quality of life. 

While PSG is the main diagnostic tool for confirming OSA, on the other hand, it does not give insight into the anatomical location of the obstruction. Hence, a presurgery patient assessment needs to be conducted to identify the potential sites causing the obstruction. There are several diagnostic methods that are used for this purpose, such as flexible fiberoptic nasopharyngoscopy, lateral cephalogram, 3D cone-beam CT scans, sleep endoscopy, and cine-MRI [36]. The latter two are especially valuable since they give insight into the dynamic aspect of the upper airway while sleeping.

### 3.1. Maxillomandibular Advancement

Maxillomandibular advancement (MMA) is typically used for the treatment of refractory or severe OSA or for those with evident and significant maxillomandibular deficiency. The advancement is performed after a Le Fort I osteotomy with a sagittal mandibular split. This consequently enhances the airway space as it pushes forward both the tongue base and the soft palate. MMA is assumed as a highly effective treatment choice for OSA linked with substantial improvements in AHI and RDI [37]. In their meta-analysis, Zaghi et al. reported that in the sample of 518 patients, they found that after MMA, 98.8% had significant improvement in outcomes regarding OSA [38]. There was a significant improvement in postoperative blood oxygen saturation and daytime sleepiness. Moreover, it was found that patients with a more severe preoperative OSA had a lower tendency for surgical success and OSA improvement. John et al. conducted a meta-analysis including details of 462 patients extracted from 20 studies [39]. They showed that, according to AHI and RDI outcomes, the surgical success of MMA was 100%.

Studies show that patients with the highest severity of OSA also have the highest degree of improvement after MMA while, on the other hand, patients with less severe OSA encounter a lesser degree of change in postoperative AHI or RDI, but they have the greatest probability of curing OSA with MMA [38]. Furthermore, patients who have high residual AHI and RDI scores are highly probable to benefit from MMA treatment [40]. MMA causes significant patient morphology change, which can be well-accepted or less favorable, such as an excessively protrusive lower face. Regarding these aesthetic and functional changes, it was advised to modify the MMA surgery technique, which led to the development of the newest surgery adjustments [39]. Modification can consist of a Le Fort I osteotomy with a segmentation or modified step design, counterclockwise or clockwise rotation of the maxillomandibular compound, and reverse T mandibular osteotomy (genioglossal advancement and advancing genioplasty) [41,42]. It is important to highlight that, in general, MMA has a good risk–benefit ratio, and successful outcomes can be achieved with minimal long-term treatment-related adverse outcomes [43]. The advantages of MMA include improved aesthetics, improvement of OSA symptoms, and correcting functional issues that ride along with malocclusion, and the disadvantages are classic surgical adverse effects, such as wound site infection and bleeding, postoperative pain, fixation plate infection, temporomandibular joint pain, limited mouth opening, and sensitive and motor nerve deficit [44].

### 3.2. Maxillary Expansion and Maxillomandibular Expansion

Patients with a narrow or high-arched hard palate (i.e., transverse maxillary deficiency) are predisposed to OSA and usually have malocclusion and dental crowding, which could be treated with rapid maxillary expansion (RME). One of the characteristics of transverse maxillary deficiency is asymmetric growth of the jaw and consequently discordant size of the maxilla in relation to the mandible. These variations of the skeleton can consequently cause a heightened nasal airflow resistance and a more posterior location of the tongue [45]. As a result of a higher nasal floor, these patients have a higher predisposition for developing nasal airway obstruction, particularly if they also have a septal deviation or enlarged inferior turbinates [46,47].

It was established that RME improves OSA in children [48]. In this population, RME is generally managed without surgery by just using orthodontic apparatus, which often has an expansion screw with numerous arms that administer forces precisely to the maxillary suture through the anchor teeth. RME creates palatal widening, flattening of the palatal arch, inferior movement of the maxilla, and change in the alignment of the mandible [49]. Machado-Júnior et al. conducted a meta-analysis regarding RME for OSA treatment in children [50]. Their outcomes showed that there is a significant decrease in AHI after RME. Moreover, this method improves nasal respiration, increases the maxillary dental arch, and improves the tongue position, and all of these effects can contribute to a reduction in AHI. Additionally, it could be hypothesized that after the broadening of the maxilla, subsequently, there is a rise in the tension of the muscles attached to the palate, which possibly has an alleviating impact on the collapsibility of the upper airway during sleep.

In their randomized controlled trial, Gokce et al. compared different kinds of rapid maxillary expansion appliances on OSA, so they divided patients into three groups: tooth tissue-borne, tooth-borne, and bone-borne expanders [51]. The AHI index did not show any significant difference between the groups; hence, their conclusion was that this is not an effective OSA treatment. 

Anatomic and functional factors contribute to the multifactorial etiology of OSA. Amid the soft tissue causes associated with upper airway anatomy, there are skeletal factors described that could have a direct influence on OSA by changing the airway space [52,53].

The treatment modality of transverse maxillary hypoplasia for adults is surgically assisted rapid maxillary expansion (SARME) or distraction osteogenesis maxillary expansion (DOME) [53]. SARME is a combination of orthodontics and surgery that creates an enlargement of the maxillary apical base and the palatal vault, supporting space for the tongue for correct swallowing. Nevertheless, there is a noticeable subjective improvement in nasal breathing associated with the enlargement of the nasal valve. This procedure has been used since 1938 and has become widely accepted as an effective and safe technique for maxillary expansion with minor complications [54,55]. Vinha et al. published an article about the SARME technique for the treatment of OSA in adult patients with good results regarding OSA symptoms, such as decreased rates of respiratory disturbances, desaturation, microarousal, and reduced daytime sleepiness [52]. There are consequently some negative outcomes connected to this surgical method, such as skeletal relapse, different kinds of periodontal problems, lack of movement or failure, gingival recession, pain sensation, and necrosis of the oral mucosa associated with the device [53] 

DOME is a method where customized distractors for each patient are first created using 3D cone-beam computed tomography (CBCT). Next, miniscrews are fixed through the palatal roof of both cortical plates as close to the midline as possible. In the last step, a LeFort I osteotomy is conducted. The expander is activated several days after the surgery by using the axial screw, and on average, approximately 8–12 mm of maxillary expansion is created [56]. Recently, Yoon et al. conducted a study on 75 subjects with a narrow maxilla and nasal floor who underwent DOME. The outcomes showed that DOME significantly relieves nasal obstruction, improves the amount of REM sleep, and decreases AHI [56]. Prior to DOME, classic surgically assisted palatal expansion techniques were used and frequently linked to major adverse effects, such as hemorrhage and a high rate of relapse. Compared to that, DOME is less aggressive and has minimal side effects, such as minimal asymmetry (which could be corrected with an orthodontic appliance) and transitory paresthesia of V2 in the anterior maxilla that would resolve in 1–6 months [57]. 

In their systematic review and meta-analysis, Abdullatif et al. reported six articles regarding maxillary expansion in adults, and they showed that the AHI in these studies has significantly decreased [46]. They also reported outcomes of two studies regarding maxillomandibular expansion which also showed a significant reduction in AHI. However, since there were only 39 patients included in these studies, further, larger-scale investigations with a larger sample should be conducted. 

### 3.3. Septoplasty, Turbinate Reduction, and Polypectomy

Nasal anomalies, such as alar collapse, nasal valve compromise, bony deformities, nasal polyps, inferior turbinate hypertrophy, and septal deviation, can boost negative intraluminal pressure, initiating airway collapse at the oropharynx or hypopharynx [58]. Procedures used to overcome nasal obstruction include septoplasty, turbinate reduction, and polypectomy. 

Septoplasty implicates adjustment of the nasal septum with a lot of different techniques that are used based on the type and the location of the deviation. Even a small reduction of the frontal deviation has been found to cause a substantial improvement of the nasal airway resistance while the restoration of a posterior deviation showed a significantly smaller effect on the airway resistance. 

Turbinate reduction is a method that decreases the dimension of the middle or inferior turbinates. The techniques used for turbinate reduction include partial or submucous resection, outfracture, superficial or submucosal cautery, laser treatment, radiofrequency treatment, and endoscopic excision of the concha bullosa [59]. Disregarding the type of nasal surgery, complications are largely the same, such as bleeding, postoperative pain and discomfort, nasal septal perforation, empty nose syndrome, chronic nasal dryness, change in the aesthetic appearance of the nose, etc. [60,61].

In their review, Cai et al. showed that septoplasty, turbinate reduction, rhinoplasty, and sinus surgery subsequently improve OSA-related quality-of-life measures and CPAP tolerance [62]. Takahashi et al. presented the results of patients who had chronic hypertrophic rhinitis and nasal septal deviation and underwent septoplasty and submucous turbinectomy [63]. The postoperative results showed a significant decrease in AHI and the awakening response index while there was also an increase in the mean blood oxygen saturation. However, on the contrary, the recent study by Migueis et al. found that after nasal surgery, there were no differences between baseline and postsurgical values in the frequency of respiratory disturbances, the total apnea time, the distribution of the apnea time within the different apnea types (obstructive and nonobstructive), or the severity of the nocturnal desaturations [64]. Equivalent to these results are outcomes by Kalam, who found that nasal surgery alone, even when nasal obstruction is a predominant symptom, may not be enough to produce a recognizable improvement [65]. These studies suggest that nasal surgeries are possibly ineffective for OSA since they do not improve events during polysomnography.

Furthermore, Hisamatsu et al. described a compound nasal surgery (CNS) method-septoplasty combined with submucosal inferior turbinectomy and posterior nasal neurectomy to assure low nasal resistance during sleep in a total of 45 patients [66]. This surgery method, therefore, inhibits mucosal swelling and nocturnal nasal resistance under a cholinergic dominant action. Regarding the allergy symptoms, it should be emphasized that the outcomes showed improvement of these symptoms in patients who had severe and moderate OSA. Furthermore, it can be concluded that from all nasal surgical procedures, the CNS method of surgery could be the most helpful in patients with severe and moderate OSA since it showed the most significant improvements in PSG findings [66].

Regarding polypectomy, Weder et al., in their literature review and case report, concluded that there are only three articles investigating the association of antrochoanal polyps in children and OSA [67]. In pediatric patients, antrochoanal polyps can be connected to daytime sleepiness and problems with concentration as OSA symptoms and require further investigation and more studies. 

### 3.4. Surgeries of the Oropharynx

The oropharynx of some patients with OSA has excessive tissue that tends to be loose and elongated. Hence, surgery of the oropharynx in the treatment of OSA aims to reduce that excessive amount of the tissue and further stiffen it. The first upper airway surgery that was employed as a treatment method for OSA was the surgery of the soft palate [68,69]. Uvulopalatopharyngoplasty (UPPP) was introduced by Fujita et al., and currently, it is, with or without tonsillectomy, the most commonly implemented surgery for OSA in adults [70]. UPPP increases the upper airway space and alleviates the collapsibility of the pharynx by partial resection of the uvula and the soft palate. It was also shown that UPPP lowers CPAP pressure requirements and consequently improves CPAP compliance in some patients [71]. However, studies showed that the success rates after sole UPPP is highly questionable, and there are several major complications related to UPPP surgery, such as edema of the upper airway, bleeding, velopharyngeal insufficiency, and nasal regurgitation [72,73,74]. On the other hand, it was established that the combination of UPPP and tonsillectomy is a highly successful surgical treatment for OSA. There are several variations of the UPPP technique as well as similar methods, such as laser-assisted uvulopalatoplasty and upper airway radiofrequency treatment [75]. Complications reported with these procedures were pain, localized edema of the tissues, mucosal sloughing, and oronasal fistulization. Recently, the results of several studies showed that radiofrequency treatment has significant success in the reduction of snoring but limited use for treating OSA [76,77,78,79]. However, another study also showed that radiofrequency tissue reduction significantly improves the tolerance and adherence of severe OSA patients using CPAP [80].

Over time, oropharyngeal surgical methods have advanced from excisional techniques towards the more preservative remodeling of the oropharynx, particularly targeting the lateral walls and conducting expansion of the velopharynx space [81]. Among the first of the aforementioned methods was transpalatal advancement pharyngoplasty (TPA), a technique introduced by Woodson et al. who intended it for patients who underwent unsuccessful UPPP and were found to have lasting velopharyngeal collapse [82]. This technique is used to widen the retropalatal space by pulling and fixating the soft palate aponeurosis forward. The results of the meta-analysis by Volner et al. have shown that TPA has significantly improved the AHI in patients with OSA [83]. Furthermore, Cahali introduced lateral pharyngoplasty (LP), a technique in which a suture is placed between the palatoglossal and superior pharyngeal muscle while the latter was previously sectioned in the vertical direction. This method splits the lateral walls of the pharynx and subsequently alleviates the collapsibility of the velopharynx and oropharynx. Furthermore, while preventing excess bleeding and maintaining optimal swallowing function are important when performing any palatopharyngoplasty, on the other hand, this method was reported with the possibility of transient dysphagia [84,85]. 

More recently, Pang and Woodson described the expansion sphincter pharyngoplasty (ESP), a method that preserves the superior pharyngeal muscle, but the palatopharyngeal muscle is split and fixated to the pterygoid hamulus [86]. This creates tension in the lateral walls of the pharynx and consequently treats both the velopharynx and oropharynx collapse and expands the retropalatal area. ESP is an alternative for patients who are not eligible for CPAP. Recently, a meta-analysis showed that ESP has great results in treating OSA and appears to have the lowest complication rates compared to other techniques [87]. 

Palate suture suspension is a more recent surgical technique for managing OSA which aims to suspend the soft palate and stabilize the lateral walls of the pharynx by using sutures for fixation. Up until now, there are several similar techniques described in the literature, all of them applying sutures for suspension [88,89,90,91,92,93,94,95]. The main aim of these techniques is pulling the soft palate forward and laterally while also achieving stabilization of lateral walls by fixing the sutures to firm structures without implementing resection, and due to this, they are considered less invasive compared to ESP or UPPP [96]. 

The Pillar implant system is a newer, minimally invasive technique for managing OSA. There are several variations of this method, but the aim is to induce local stiffness and prevent soft palate collapse in the state of muscle relaxation during sleep. That is achieved by the insertion of artificial implants in the soft palate which significantly alleviates snoring and treats OSA [97]. Disadvantages are foreign body sensation, swallowing difficulties as well as risk for ulceration and implant extrusion. Zhang et al. have described a method in which “U-shaped” titanium mesh and titanium screws are used as implants that are positioned into a soft palate [98]. Its short-term efficacy is good in patients with moderate-to-severe OSA and velopharyngeal obstruction.

### 3.5. Genioglossal Advancement

Genioglossal advancement (GA) was first described by Riley et al., and it is indicated for patients with a narrow lower pharynx [99]. The procedure involves repositioning the genial tubercle and genioglossus muscle forward, thus enlarging the airway in the anteroposterior dimension. The osteotomy extends over the mandible to incorporate the genioglossus attachment while also leaving the dentoalveolar process intact. The main disadvantages of this procedure were the possibility of mandibular fracture, bony segment necrosis, and a risk of lower incisor teeth damage, so several variations were proposed to improve results and reduce complications [100,101,102,103]. They include diverse forms of osteotomies and minor modifications in the transposition of the bony segment. Li et al. presented an alteration of the procedure making a “window” osteotomy by leaving an inferior border of the mandible intact, thus reducing the potential of mandibular fractures [104]. Garcia Vega et al. presented a modified GA technique that reduces muscle damage but is more complicated to perform, especially on smaller mandibles [100]. Nevertheless, the main aim of the technique is the same, and outcomes have shown significant improvement in OSA. A recent systematic review by Kezirian et al. reported a surgical success rate of 67% when undergoing GA as a sole treatment [105]. The recent development of virtual simulation surgeries using 3D technology has helped surgeons to plan more precise osteotomies, thus minimizing the complications of conventional GA procedures [106]. 

### 3.6. Hyoid Suspension

Forward movement of the hyoid complex can improve the hypopharyngeal airway due to the excessively low position of the hyoid bone in patients with OSA. Riley et al. described the original method for hyoid myotomy with suspension, and it involved an inferior hyoid myotomy of the infrahyoid musculature followed by suspension of the hyoid to the mandible using fascia lata [107,108]. Due to high morbidity, the technique was later modified to move the hyoid bone forward inferiorly over the thyroid cartilage which is known as hyoidthyroidpexia [109]. The goal of this modification is to increase the retrolingual airway space [110]. Most recent alterations of this treatment include using a stainless steel wire or titanium plates on the thyroid cartilage, thus preventing its damage [111,112]. The use of implantable devices for hyoid expansion was also reported, but no significant improvements in sleep parameters were noted [113]. Serious postoperative complications were rarely reported, and the most common ones were dysfunctions of pronunciation and swallowing [114]. A meta-analysis by Song et al. showed that sole hyoid surgery can reduce OSA severity in adults [115]. Their outcomes showed a significant decrease in sleepiness and that AHI was reduced by 38% whereas for hyothyroidopexy, an AHI reduction of 50.7% was achieved.

### 3.7. Tongue Base Reduction

Base of tongue (BOT) reduction may be performed through several approaches, such as radiofrequency, surgery, endoscopy, and transoral robotic surgery (TORS) [116]. Radiofrequency surgery has limited indications and is mostly used in cases of moderate tongue base hypertrophy [117]. Midline glossectomy (MLG) involves removing parts of the middle and back of the tongue (usually using a laser) [118]. It is mostly combined with other surgical procedures, such as UPPP, palatoplasty, palatopharyngoplasty, or epiglottidectomy. Submucosal minimally invasive lingual excision (SMILE) is a technique that avoids damaging the mouth floor muscles while undergoing excision of excessive tongue tissue using an endoscope and ultrasound for visualization [119]. A meta-analysis by Murphey et al. showed improvement in outcomes when glossectomy is a part of multilevel surgery in adults with OSA [120]. There are not many reports on the effect of glossectomy as a single treatment, but the study by Suslu et al. showed that coblation MLG is a beneficial procedure when carried out solely with a success rate of 52% [121]. Moreover, it was shown that MLG was very successful when implemented solely in patients who failed UPPP. 

With the improvement of new technologies, TORS has become a therapeutic alternative for reducing tongue volume. It is useful due to an endoscope that enables a 3D visualization of the surgical field and a robotic instrument that is handled to reduce the tongue base in an area that is usually very difficult to access through classical surgical methods. The reported complications are bleeding, dehydration, dysphagia, oropharyngeal stenosis, tongue numbness, and dysgeusia [114,122]. In the study by Friedman et al., a comparison of the outcomes between TORS, SMILE, and radiofrequency BOT reduction was conducted. It was shown that TORS in combination with palatopharyngoplasty had a significantly higher reduction of AHI scores [123]. Hwang et al. compared TORS with an endoscope-guided coblation BOT reduction and found that there were no significant differences regarding the outcomes [124]. Moreover, the results of the meta-analysis by Lechien et al. suggest that TORS BOT reduction is associated with improvements in AHI and ESS irrespective of any additional procedures performed [125].

### 3.8. Tongue Base Suspension

The tongue base suspension (TBS) is a minimally invasive surgical technique whose goal is to reduce the tongue base collapse and enlarge the retrolingual airway [126]. A standard procedure is carried out using a Repose system (Repose Surgical Kit, CKA Air Vance, Medtronic, Inc.). Through a submental incision, a screw is placed into the mandible, and the tongue is stabilized with a suture using a screw as an anchor [127]. Hsin et al. most recently presented a transoral tongue base suspension (TOTS) method [128]. Its main advantage is using an intraoral vestibular incision and no need for screws. Even though there are modifications of the procedure reported, TBS using a Repose system remains a gold standard [129]. It was shown that it has a cure rate of 81.8% when combined with UPPP in patients with multilevel airway obstruction, and it is a good alternative for other more invasive operative procedures, such as GA or various BOT techniques [130]. The most commonly reported complications have been sialadenitis, tongue base swelling, infection, or suture-related problems [131].

### 3.9. Adenoidectomy and Tonsillectomy

A tonsillectomy is a common surgical treatment used for tonsillar infections and inflammations or tonsillar malignant diseases. However, studies have shown that there is an association between the volume and grade of the tonsils with the AHI and the severity of OSA, but only a few studies have discussed isolated tonsillectomy as a surgical treatment for treating OSA in adults [132,133,134,135,136]. Still, it is important to highlight that the outcomes of several studies imply an association between tonsillar hypertrophy and OSA, and this suggests that tonsillectomy alone is an effective treatment for OSA in adults with tonsil hypertrophy [133,137]. It is considered a simple procedure with few complications, such as temporary pain and dysphagia. Several studies showed that a tonsillectomy considerably reduced the AHI and improved daytime sleepiness and the severity of OSA in these patients [134,138]. Nevertheless, for the management of OSA, tonsillectomy is more commonly applied in combination with UPPP [139,140]. Evidence is showing that the efficacy of UPPP is significantly amplified when joined with a tonsillectomy, and studies established that it leads to an additional improvement of the AHI, snoring, and daytime sleepiness [141]. Regarding pediatric OSA, adenotonsillectomy is the first line of treatment [142]. It is suggested as a safe day-case surgery in carefully selected patients [143]. The results of most secondary analyses suggest that children who underwent adenotonsillectomy experienced the greatest improvements in symptom burden, sleepiness, parent-reported behavior, and quality of life.

### 3.10. Tracheostomy

A tracheostomy is a procedure that involves creating an opening in the trachea to bypass the upper airway. It was first introduced as a treatment for OSA in 1969, and until then, there were no other surgical options for treating OSA [144]. Since patients with a tracheostomy avoid the whole upper airway, it is the most effective surgical procedure for treating OSA [145]. However, it was shown that patients with concomitant cardiopulmonary diseases or chronic obstructive pulmonary diseases may have residual indispositions [146]. A systematic review by Camacho et al. found that a tracheostomy substantially reduces the AHI and daytime sleepiness while also improving oxygen desaturation index and cardiovascular mortality in patients with OSA [147]. Nevertheless, a permanent tracheostomy tube requires delicate care, and it restricts daily living activities and social interaction. Hence, it should be used as a treatment for OSA only in carefully chosen patients who do not have other surgical options. On the other hand, a temporary tracheostomy can be performed on patients as a preliminary prophylactic measure. Presently, tracheostomy is mostly retained for patients who are not eligible to use CPAP and/or do not have a verifiable upper airway abnormality [148,149].

## 4. Conclusions

There are a lot of surgical methods and modalities for treating OSA, and they all have advantages and disadvantages. Even though, according to the literature, some surgical methods seem more successful, on the other hand, due to the fact that the anatomical causes of OSA are very heterogeneous, every patient should be approached individually, and the best surgical method should be chosen for his specific needs.

Regarding the possibility that surgical therapy from the OMFS domain could be a trigger for developing OSA, the literature is still too scarce on this subject, and only lately more attention has been drawn to this issue. Nevertheless, the results of several recent studies are implying that oncology, traumatology, and orthognathic surgery could really be impacting OSA. Henceforth, further studies, especially systematic reviews, are needed regarding this important subject. 

## Data Availability

Not applicable.
